# The Evolving Landscape of Pulmonary Vascular Disease: Current Advances and Future Directions in Pulmonary Hypertension and Pulmonary Embolism

**DOI:** 10.3390/life16081364

**Published:** 2026-08-19

**Authors:** Michele Antonio Cacia, Egidio Imbalzano, Marco Vatrano

**Affiliations:** 1 Cardiology Unit, “Renato Dulbecco” University Hospital, “Pugliese” Hospital, 88100 Catanzaro, Italy; 2 Department of Clinical and Experimental Medicine, University of Messina, 98125 Messina, Italy; egidio.imbalzano@unime.it

## 1. Introduction

**Figure 1:**
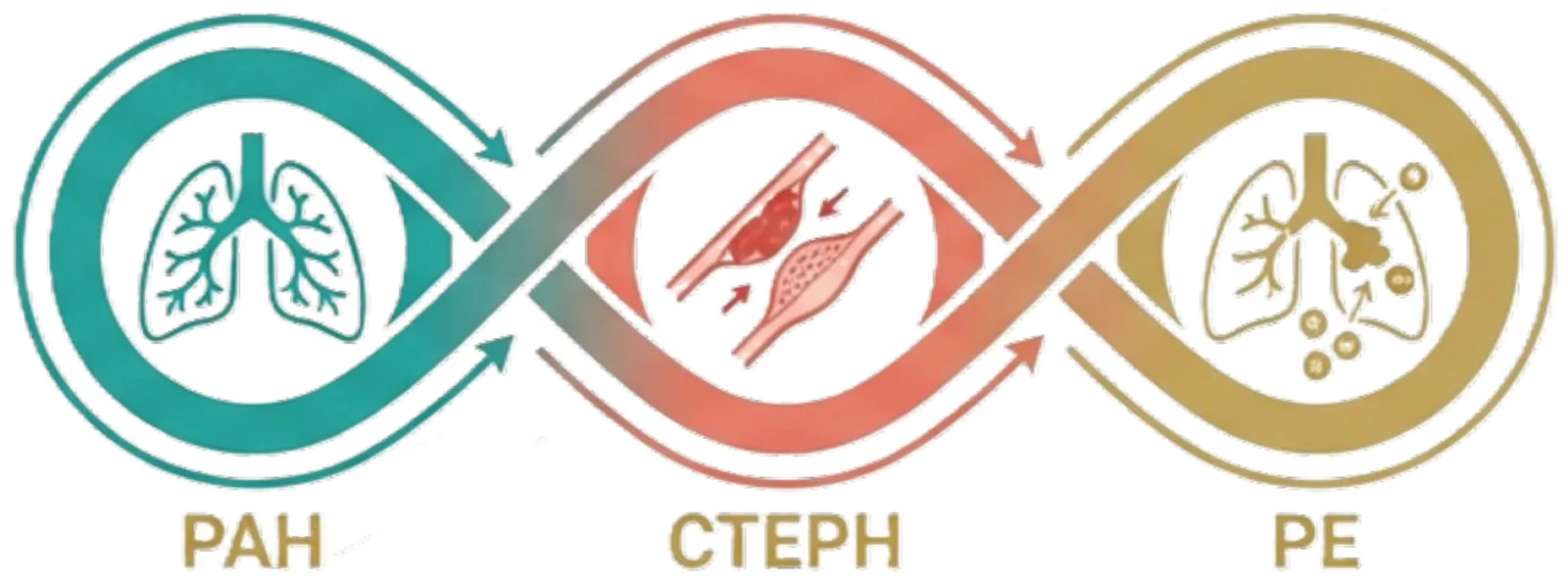
The continuum of pulmonary vascular disease.

Pulmonary vascular diseases have undergone a profound transformation over the past two decades. Once characterized by delayed diagnosis, limited therapeutic options, and poor prognosis, pulmonary hypertension (PH) and pulmonary embolism (PE) are now managed through increasingly mechanism-based and personalized approaches driven by advances in molecular biology, cardiovascular imaging, invasive hemodynamics, pharmacotherapy, and catheter-based interventions (Table 1). PH is currently recognized as a heterogeneous syndrome characterized by progressive pulmonary vascular dysfunction and elevated pulmonary arterial pressure [1], affecting approximately 1% of the general population and nearly 10% of individuals older than 65 years [2]. Beyond its hemodynamic definition, PH results from complex interactions among endothelial dysfunction, inflammation, aberrant vascular cell proliferation, metabolic dysregulation, thrombosis, and maladaptive right ventricular remodeling, ultimately leading to increased pulmonary vascular resistance and right ventricular failure, the major determinant of prognosis [3]. Similarly, the understanding of PE has evolved from an isolated thromboembolic event to a spectrum of pulmonary vascular disease that may progress to persistent vascular obstruction, chronic thromboembolic pulmonary hypertension (CTEPH), and long-term functional impairment. This paradigm shift has fostered more refined diagnostic algorithms, risk stratification models, and reperfusion strategies [4]. The 2022 ESC/ERS Guidelines [5] marked a major advance by redefining the hemodynamic criteria for PH (mean pulmonary arterial pressure >20 mmHg, pulmonary arterial wedge pressure ≤15 mmHg, and pulmonary vascular resistance >2 Wood units for pulmonary arterial hypertension) and emphasizing early diagnosis and multidimensional risk assessment before irreversible vascular remodeling occurs. Therapeutic strategies have likewise evolved from predominantly vasodilator-based treatment toward disease-modifying interventions targeting key molecular pathways involved in pulmonary vascular remodeling [6], including transforming growth factor-β (TGF-β) and bone morphogenetic protein receptor type 2 (BMPR2) signaling [7]. Concurrently, CTEPH [8] management has become increasingly multidisciplinary, integrating pulmonary endarterectomy, balloon pulmonary angioplasty, targeted pharmacotherapy, advanced imaging, and specialized referral networks, while acute PE treatment has benefited from catheter-directed therapies and the implementation of Pulmonary Embolism Response Teams (PERTs). These advances support a unified view of PH and PE as interconnected manifestations of pulmonary vascular disease (Figure 1) sharing common mechanisms of endothelial dysfunction, thrombosis, vascular remodeling, inflammation, and right ventricular adaptation [9]. Recognizing these shared biological pathways provides the foundation for integrated diagnostic and therapeutic strategies across the spectrum of pulmonary vascular disorders.

## 2. Pulmonary Arterial Hypertension: From Pathobiology to Modern Therapy

Pulmonary arterial hypertension (PAH) is a progressive vasculopathy characterized by complex and interconnected biological processes that ultimately culminate in right ventricular failure. Its contemporary management reflects a continuous evolution from empiric vasodilation toward mechanism-driven and risk-stratified therapeutic strategies. This section explores the key pathogenic pathways and therapeutic milestones that have shaped current clinical practice, highlighting the transition from traditional vasodilator-based approaches to emerging disease-modifying and precision medicine strategies.

### 2.1. The Evolution of Disease Understanding

Over the past three decades, PAH has evolved from being regarded as a predominantly vasoconstrictive disorder to a complex proliferative vasculopathy characterized by progressive remodeling of the pulmonary arterial circulation. Histopathological hallmarks include endothelial dysfunction, medial hypertrophy, intimal fibrosis, adventitial thickening, inflammatory infiltrates, and plexiform lesions, collectively leading to increased pulmonary vascular resistance and right ventricular failure [10]. A breakthrough in PAH pathobiology was the identification of BMPR2 mutations, present in over 80% of heritable and approximately 20% of idiopathic cases. BMPR2 signaling plays a central role in maintaining pulmonary vascular homeostasis by limiting smooth muscle proliferation and preserving endothelial integrity. Its disruption promotes uncontrolled vascular remodeling and disease progression. Concurrently, chronic inflammation has emerged as a key driver of PAH, with elevated circulating cytokines—including interleukin-6, interleukin-1β, and tumor necrosis factor-α—consistently associated with disease severity and adverse outcomes [11]. More recently, mitochondrial dysfunction, metabolic reprogramming, epigenetic regulation, and immune dysregulation have further expanded the biological framework of PAH, shifting therapeutic strategies from symptom-oriented vasodilation toward interventions targeting the molecular mechanisms underlying vascular remodeling.

### 2.2. The Classical Therapeutic Triad

Pharmacological management of PAH has been based on targeting three key signaling pathways involved in the regulation of pulmonary vascular tone and remodeling: the endothelin, nitric oxide, and prostacyclin pathways:

- Endothelin pathway. Endothelin-1 is a potent vasoconstrictor and mitogen that contributes to pulmonary vascular remodeling. Endothelin receptor antagonists (ERAs), including bosentan [12], ambrisentan [13], and macitentan, improve exercise capacity, functional status, and hemodynamics while reducing clinical worsening. The SERAPHIN trial [14] established macitentan as a cornerstone of long-term therapy by demonstrating a significant reduction in morbidity and mortality events.
- Nitric oxide pathway. Nitric oxide promotes vasodilation through soluble guanylate cyclase activation and cyclic guanosine monophosphate (cGMP) signaling. Phosphodiesterase-5 inhibitors, such as sildenafil [15] and tadalafil [16], enhance endogenous NO activity by preventing cGMP degradation, improving pulmonary hemodynamics and exercise capacity. Riociguat, a soluble guanylate cyclase stimulator, directly increases cGMP production independently of endogenous NO availability, providing an alternative mechanism particularly relevant in endothelial dysfunction and establishing its role in both PAH and inoperable or persistent CTEPH [17].
- Prostacyclin pathway. Prostacyclin exerts potent vasodilatory, antiproliferative, anti-inflammatory, and antiplatelet effects that are markedly reduced in PAH. Intravenous epoprostenol remains the only therapy to demonstrate a survival benefit in randomized trials [18], while treprostinil [19], iloprost [20], and the selective IP receptor agonist selexipag [21] have expanded treatment options across different stages of disease.

### 2.3. Sotatercept and the Emergence of Disease-Modifying Therapy

Although therapies targeting the endothelin, nitric oxide, and prostacyclin pathways have substantially improved clinical outcomes in PAH, their primary effects remain centered on vasodilation and symptom control rather than reversal of the underlying vasculopathy. Advances in the understanding of pulmonary vascular biology identified dysregulation of the transforming growth factor-β (TGF-β)/bone morphogenetic protein receptor type 2 (BMPR2) signaling axis as a central mechanism driving vascular remodeling [22]. Excessive activin signaling, together with impaired BMPR2-mediated antiproliferative pathways, promotes abnormal smooth muscle proliferation, endothelial dysfunction, and progressive pulmonary vascular remodeling. Sotatercept, a first-in-class activin receptor type IIA ligand trap, was developed to restore this signaling imbalance by sequestering circulating activins and growth differentiation factors, thereby re-establishing the balance between pro- and antiproliferative pathways. Unlike conventional vasodilators, sotatercept directly targets the molecular mechanisms underlying disease progression. Across phase II [23] and III [24] clinical trials, sotatercept significantly improved exercise capacity, pulmonary hemodynamics, right ventricular load, biomarkers of cardiac stress, functional status, and time to clinical worsening, with consistent benefits observed on top of optimized background therapy. The introduction of sotatercept represents a paradigm shift in PAH management, marking the transition from exclusively vasodilator-based treatment toward disease-modifying therapy. Although long-term experience is still limited, its clinical success has established proof of concept that targeting pulmonary vascular remodeling is feasible and may redefine future therapeutic strategies in pulmonary arterial hypertension.

### 2.4. Combination Therapy and Contemporary Risk Stratification

The management of PAH has evolved from sequential monotherapy toward an upfront, risk-oriented combination strategy [25]. This paradigm shift reflects the recognition that the endothelin, nitric oxide, and prostacyclin pathways contribute simultaneously to disease progression, making single-pathway inhibition insufficient for optimal disease control. The AMBITION trial [26] established initial combination therapy with ambrisentan and tadalafil as the preferred treatment strategy for most newly diagnosed patients by significantly reducing the risk of clinical failure compared with monotherapy. Subsequent evidence from the SERAPHIN and GRIPHON^21^ trials further demonstrated that comprehensive pathway inhibition reduces morbidity and delays clinical worsening, supporting combination therapy as the standard of care. Current management is guided by serial multidimensional risk assessment, with the primary therapeutic goal of achieving and maintaining a low-risk profile [27]. Contemporary risk models integrate clinical status, exercise capacity, circulating biomarkers, right ventricular function, and invasive hemodynamic parameters [28]. Accordingly, the 2022 ESC/ERS Guidelines recommend reassessment every 3–6 months, with prompt treatment escalation whenever low-risk status is not achieved. Risk stratification continues to evolve with the incorporation of more refined markers of right ventricular performance. Among these, stroke volume index has emerged as a promising prognostic parameter, providing incremental information beyond conventional cardiac output measurements and complementing advanced imaging techniques such as cardiac magnetic resonance [29]. The integration of imaging, biomarkers, and hemodynamic profiling is progressively enabling a more individualized approach to disease assessment and therapeutic decision making.

### 2.5. Precision Medicine and Phenotypic Diversity in PAH

Although PAH is traditionally classified according to etiology, growing evidence indicates that patients with similar clinical and hemodynamic profiles may exhibit distinct molecular mechanisms, treatment responses, and prognoses [30]. This biological heterogeneity has fueled the transition toward precision medicine, in which therapeutic strategies are increasingly tailored to individual disease phenotypes. Genetic profiling has been central to this evolution. Beyond the pivotal role of BMPR2, pathogenic variants in genes involved in vascular development and signaling—including ACVRL1, ENG, SMAD9, TBX4, KCNK3, and EIF2AK4—have further highlighted the genetic complexity of PAH and may contribute to future risk stratification and treatment selection [31]. Similarly, inflammatory and immune-mediated mechanisms have emerged as important modulators of pulmonary vascular remodeling. Elevated circulating cytokines and perivascular inflammatory infiltrates support the existence of distinct immune phenotypes that may become targets for future immunomodulatory therapies. Among emerging clinical phenotypes, reduced diffusing capacity for carbon monoxide (DLCO) [32] has been associated with a more severe disease profile characterized by higher pulmonary vascular resistance, poorer functional status, and a diminished response to conventional vasodilator therapy. These observations suggest that DLCO may serve as a practical surrogate marker of pulmonary vascular phenotype and therapeutic responsiveness. Advances in multimodality imaging and biomarker discovery are further accelerating this transition. Cardiac magnetic resonance has become the reference standard for right ventricular assessment [33], while emerging techniques—including myocardial strain, tissue characterization, and four-dimensional flow imaging—provide increasingly refined evaluation of right ventricular adaptation and ventricular–arterial coupling. In parallel, developments in proteomics, metabolomics [34], transcriptomics, and artificial intelligence are expected to identify novel biomarkers capable of improving prognostic assessment and predicting treatment response. Collectively, these advances indicate that the future management of PAH will move beyond conventional clinical classification toward integrated phenotypic characterization combining genetics, imaging, biomarkers, physiology, and molecular profiling to enable truly personalized therapy.

## 3. Chronic Thromboembolic Pulmonary Hypertension: A Multimodal Therapeutic Paradigm

CTEPH occupies a unique position within the spectrum of pulmonary vascular diseases, representing the only potentially curable form of PH. Over the past decade, advances in diagnosis, surgical techniques, catheter-based interventions, and targeted pharmacotherapy have transformed CTEPH from a frequently underrecognized disorder into a model of multidisciplinary management. Although estimated to affect 14–144 individuals per million population, its true prevalence is likely underestimated owing to delayed diagnosis and under-recognition [35].

### 3.1. Pathophysiology: Beyond Persistent Thromboembolic Obstruction

CTEPH is no longer considered simply the consequence of unresolved pulmonary emboli. Current evidence indicates that the disease arises from the interplay between persistent organized thrombotic obstruction of the proximal pulmonary arteries and progressive remodeling of the distal pulmonary microvasculature. Following acute pulmonary embolism, incomplete thrombus resolution may lead to fibrotic incorporation of thrombotic material into the arterial wall, while abnormal flow dynamics and increased shear stress promote endothelial dysfunction, inflammation, and secondary arteriolar remodeling resembling that observed in pulmonary arterial hypertension. This dual pathological substrate—proximal mechanical obstruction combined with distal small-vessel disease—explains the marked heterogeneity of hemodynamic impairment and accounts for persistent pulmonary hypertension in some patients despite technically successful intervention [36].

### 3.2. Pulmonary Endarterectomy

Pulmonary endarterectomy (PEA) remains the treatment of choice for patients with operable CTEPH and the only intervention capable of achieving complete hemodynamic normalization [37]. Performed in experienced centers under deep hypothermic circulatory arrest, PEA is associated with operative mortality below 5% and five-year survival exceeding 85–90% [38]. Beyond relieving vascular obstruction, successful surgery promotes substantial right ventricular reverse remodeling, improving ventricular function, exercise capacity, and long-term prognosis [39]. Nevertheless, approximately one-third of patients are unsuitable for surgery because of distal disease distribution, significant comorbidities, or an unfavorable risk–benefit profile, while others experience persistent or recurrent pulmonary hypertension after endarterectomy, highlighting the need for complementary therapeutic strategies.

### 3.3. Balloon Pulmonary Angioplasty

Balloon pulmonary angioplasty (BPA) has emerged as the preferred interventional option for patients with inoperable CTEPH or residual pulmonary hypertension after PEA [40]. Technical refinements in imaging, guidewire technology, and staged procedural strategies have markedly improved its safety profile. Contemporary studies consistently demonstrate significant reductions in pulmonary artery pressure and pulmonary vascular resistance, accompanied by improvements in exercise capacity, quality of life, and right ventricular remodeling [41]. The introduction of BPA has substantially expanded treatment opportunities, reinforcing the concept that CTEPH management should be individualized rather than based on a single therapeutic modality.

### 3.4. Targeted Pharmacological Therapy

Recognition of the contribution of distal microvascular remodeling has provided the rationale for targeted medical therapy. Riociguat remains the only approved pharmacological treatment for patients with inoperable CTEPH or persistent pulmonary hypertension after PEA, improving pulmonary vascular resistance, functional capacity, WHO functional class, and clinical outcomes through stimulation of the nitric oxide–soluble guanylate cyclase–cyclic GMP pathway [42]. Increasingly, medical therapy is integrated with surgery and BPA as bridging treatment before intervention, adjunctive therapy after revascularization, or long-term management of residual disease. This multimodal strategy has become the cornerstone of contemporary CTEPH care.

### 3.5. Future Perspectives

Future advances are expected to focus on earlier diagnosis, optimized treatment sequencing, and more precise characterization of pulmonary vascular remodeling. Artificial intelligence-assisted interpretation of ventilation–perfusion imaging, advanced multimodality imaging, machine-learning prediction models, and novel molecular biomarkers may improve patient selection and facilitate earlier intervention before irreversible right ventricular dysfunction develops [43]. The integration of surgery, catheter-based intervention, targeted pharmacotherapy, and precision diagnostics is progressively redefining CTEPH management, establishing this disease as one of the most successful examples of personalized and multidisciplinary care in pulmonary vascular medicine.

## 4. Acute Pulmonary Embolism: From Anticoagulation to Precision Reperfusion

Acute PE remains one of the leading cardiovascular emergencies, with an annual incidence of 39–115 cases per 100,000 individuals and persistently high mortality, particularly in untreated patients [44]. However, its management has evolved beyond anticoagulation toward a precision-based approach integrating clinical probability assessment, multimodality imaging, biomarkers, risk stratification, and individualized reperfusion strategies.

### 4.1. Diagnostic Advances and Contemporary Risk Stratification

Because the clinical presentation of PE is highly variable and often nonspecific, structured diagnostic algorithms are essential. Clinical prediction models, particularly the Wells score, combined with age-adjusted D-dimer testing, improve diagnostic accuracy while reducing unnecessary imaging [45]. Computed tomography pulmonary angiography (CTPA) remains the diagnostic gold standard, although emerging technologies—including dual-energy CT [46], advanced perfusion imaging, and artificial intelligence-assisted image analysis—are expected to further enhance diagnostic precision. Risk stratification represents the cornerstone of acute PE management, guiding decisions regarding reperfusion therapy and level of care. Important differences exist between the current European and recent American frameworks. The 2019 ESC guidelines maintain the traditional stratification into high, intermediate (intermediate-high and intermediate-low), and low-risk PE, with a strong emphasis on right ventricular dysfunction as a determinant of early escalation strategies. In contrast, the 2026 AHA/ACC/ACCP/ACEP/CHEST guidelines [47] introduce a revised five-tier clinical classification (A–E), designed to integrate symptom burden, hemodynamic status, and predicted risk of clinical deterioration into a more granular decision-making framework. This new system further promotes early identification of low-risk patients potentially suitable for expedited discharge, while refining the selection of candidates for advanced therapies, including catheter-based interventions and mechanical circulatory support. Despite these differences in classification structure, both European and American guidelines converge on the central principle that management should be driven by early risk stratification combining clinical assessment, imaging findings, and biomarkers, enabling timely identification of patients who may benefit from reperfusion strategies.

### 4.2. Reperfusion Strategies

Systemic thrombolysis remains the first-line reperfusion strategy for high-risk pulmonary embolism, providing rapid reduction in thrombotic burden and right ventricular afterload. However, its use is constrained by a clinically relevant risk of major bleeding, including intracranial hemorrhage, particularly in elderly patients and those with comorbidities. Both the 2019 ESC guidelines and the 2026 American guidelines (AHA/ACC/ACCP/ACEP/CHEST/SCAI) endorse systemic thrombolysis as the standard of care in hemodynamically unstable PE, with early administration in the absence of absolute contraindications. However, the American framework places greater emphasis on individualized bleeding risk assessment and introduces a more explicit role for mechanical reperfusion strategies in selected high-risk but bleeding-complicated patients. These limitations of systemic therapy have driven the expansion of catheter-based reperfusion techniques. Ultrasound-assisted catheter-directed thrombolysis allows local delivery of reduced-dose fibrinolytics, achieving improvements in right ventricular function and pulmonary hemodynamics while potentially lowering systemic bleeding risk [48]. Mechanical thrombectomy has further expanded the therapeutic armamentarium by enabling thrombus extraction without fibrinolytic administration, representing a particularly attractive option in patients with absolute or relative contraindications to thrombolysis [49]. Devices such as the FlowTriever [50] and Indigo aspiration systems [51] have demonstrated rapid hemodynamic and clinical improvement in observational studies and single-arm trials, including reductions in right ventricular dilatation and improvement in pulmonary artery pressures. However, both ESC and US guidelines emphasize that high-quality randomized comparative evidence remains limited, and therefore patient selection should be individualized and ideally guided by multidisciplinary PERTs [52]. Overall, while ESC 2019 guidelines and AHA/ACC 2026 recommendations differ in the degree of procedural emphasis, they converge on a central paradigm: reperfusion therapy in PE is no longer limited to systemic thrombolysis but now encompasses a spectrum of escalating strategies ranging from pharmacologic to catheter-directed and mechanical approaches, tailored to risk profile, bleeding risk, and institutional expertise. Routine reperfusion is not recommended in intermediate-high-risk PE; however, emerging randomized evidence indicates that catheter-directed thrombolysis may reduce early hemodynamic decompensation without a significant increase in major bleeding, although a survival benefit has not been demonstrated [53].

### 4.3. Pulmonary Embolism Response Teams

The growing complexity of PE management has led to the widespread adoption of PERTs, multidisciplinary teams that integrate expertise from cardiology, pulmonology, intensive care, hematology, interventional radiology, and cardiac surgery. PERT programs facilitate rapid risk assessment, individualized treatment selection, and timely access to advanced reperfusion therapies [54]. Although randomized data remain scarce, observational studies suggest improved implementation of catheter-based interventions together with favorable clinical outcomes, supporting PERTs as a cornerstone of contemporary PE care.

## 5. Future Perspectives: Toward Precision Pulmonary Vascular Medicine

Pulmonary vascular medicine is entering an era of precision therapeutics driven by advances in molecular biology, multimodality imaging, artificial intelligence, and systems medicine. The success of sotatercept has established proof of concept that pulmonary vascular remodeling can be therapeutically targeted, opening the way for future interventions focused on BMPR2 restoration, TGF-β modulation, inflammation, epigenetic regulation, and metabolic reprogramming. At the same time, artificial intelligence and machine-learning algorithms are expected to improve disease detection, imaging interpretation, risk prediction, and treatment selection through integration of clinical, imaging, and molecular data. Complementary advances in genomics, transcriptomics, proteomics, metabolomics, and other multi-omics technologies will likely redefine disease classification according to biological signatures rather than conventional clinical phenotypes. Finally, increasing attention is being directed toward the right ventricle as a therapeutic target. Novel strategies aimed at preserving right ventricular metabolism, limiting fibrosis and inflammation, and optimizing ventricular–arterial coupling may further improve long-term outcomes. Together with wearable technologies and remote physiological monitoring, these developments are expected to enable earlier intervention and increasingly personalized disease management.

## 6. Conclusions

**Figure 2:**
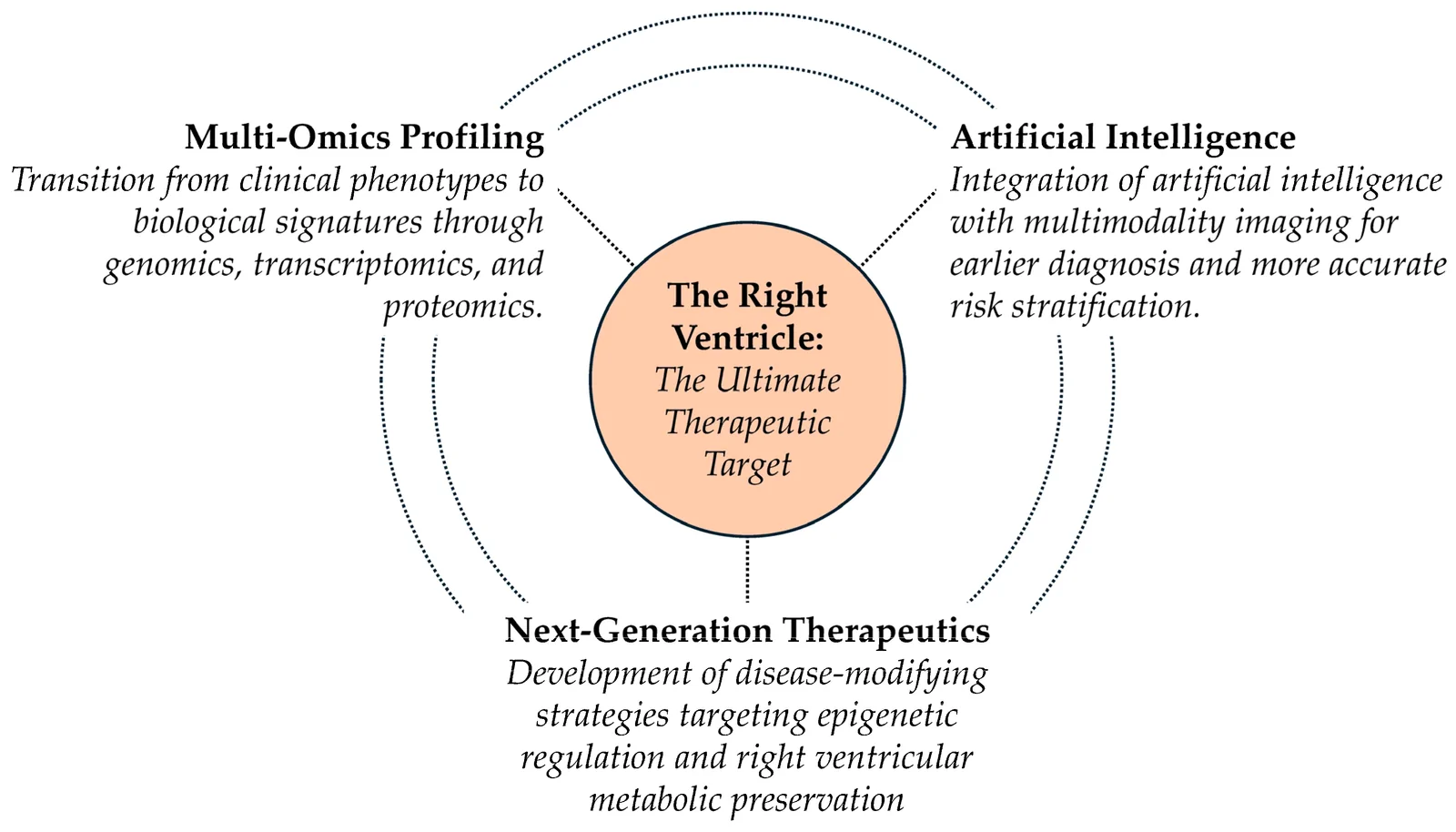
Preservation and recovery of right ventricular function, recognized as the central determinant of prognosis in pulmonary vascular disease.

Over the past two decades, pulmonary vascular medicine has undergone a profound transformation. In PAH, therapeutic strategies have evolved from vasodilator-based treatment toward disease modification through targeted manipulation of molecular pathways involved in vascular remodeling. In CTEPH, the integration of pulmonary endarterectomy, balloon pulmonary angioplasty, and targeted pharmacological therapy has established a highly effective multimodal treatment paradigm. Similarly, acute PE management has progressed from routine anticoagulation to individualized reperfusion strategies supported by advanced imaging, structured risk stratification, catheter-based interventions, and multidisciplinary PERT programs. Collectively, these advances support a unified view of PH and PE as interconnected manifestations of pulmonary vascular disease sharing common mechanisms of endothelial dysfunction, thrombosis, inflammation, vascular remodeling, and right ventricular adaptation. Future progress will increasingly depend on integrating molecular biology, precision medicine, advanced imaging, artificial intelligence, and multidisciplinary care to modify disease progression, preserve right ventricular function, and improve long-term outcomes across the spectrum of pulmonary vascular disorders (Figure 2).

## Figures and Tables

**Table 1:** Paradigm shift in pulmonary vascular medicine.

	Historical View → Contemporary Paradigm
**Focus**	Isolated thromboembolic events and vasoconstrictive disorders **→** Interconnected spectrum of progressive pulmonary vascular disease
**Diagnostic Approach**	Delayed detection, heavily reliant on late-stage symptoms **→** Early detection through multimodality imaging and multidimensional risk assessment
**Therapeutic Strategy**	Empiric vasodilation and symptom-based management **→** Mechanism-based, disease-modifying targeted therapies
**Clinical Outcome**	High morbidity, limited therapeutic options, poor prognosis **→** Multidisciplinary care models, reversed vascular remodeling, and prolonged survival
